# Orientation Cues for High-Flying Nocturnal Insect Migrants: Do Turbulence-Induced Temperature and Velocity Fluctuations Indicate the Mean Wind Flow?

**DOI:** 10.1371/journal.pone.0015758

**Published:** 2010-12-29

**Authors:** Andy M. Reynolds, Don R. Reynolds, Alan D. Smith, Jason W. Chapman

**Affiliations:** 1 Rothamsted Research, Harpenden, United Kingdom; 2 Natural Resources Institute, University of Greenwich, Chatham, United Kingdom; Max-Planck Institute of Neurobiology, Germany

## Abstract

Migratory insects flying at high altitude at night often show a degree of common alignment, sometimes with quite small angular dispersions around the mean. The observed orientation directions are often close to the downwind direction and this would seemingly be adaptive in that large insects could add their self-propelled speed to the wind speed, thus maximising their displacement in a given time. There are increasing indications that high-altitude orientation may be maintained by some intrinsic property of the wind rather than by visual perception of relative ground movement. Therefore, we first examined whether migrating insects could deduce the mean wind direction from the turbulent fluctuations in temperature. Within the atmospheric boundary-layer, temperature records show characteristic ramp-cliff structures, and insects flying downwind would move through these ramps whilst those flying crosswind would not. However, analysis of vertical-looking radar data on the common orientations of nocturnally migrating insects in the UK produced no evidence that the migrants actually use temperature ramps as orientation cues. This suggests that insects rely on turbulent *velocity* and *acceleration* cues, and refocuses attention on how these can be detected, especially as small-scale turbulence is usually held to be directionally invariant (isotropic). In the second part of the paper we present a theoretical analysis and simulations showing that velocity fluctuations and accelerations felt by an insect are predicted to be anisotropic even when the small-scale turbulence (measured at a fixed point or along the trajectory of a fluid-particle) is isotropic. Our results thus provide further evidence that insects do indeed use turbulent velocity and acceleration cues as indicators of the mean wind direction.

## Introduction

Many insect species undertake long-range, high-altitude nocturnal migrations, but one characteristic feature of these flights, at least in the larger species (such as moths or grasshoppers), has puzzled researchers for over 40 years, namely: how do insects maintain wind-related orientation at altitudes of several hundreds of metres in the dark? The considerable degree of uniformity often found in migrants' headings – behaviour known as ‘common orientation’ – was evident from the earliest radar studies [1]–[3], and it was also found that the orientating insects commonly formed layers at particular altitudes in the atmosphere. The observed orientation directions can show quite small angular dispersions (∼15°) around the mean and, in wind speeds higher than the migrants' self-propelled flight speed (air-speed), are often close to the downwind direction (or have a large downwind component) [2], [4]. This behaviour seems to be adaptive in that large insects can add their air-speed to the wind speed, thus maximizing their displacement distance in a given time.

The observed aerial densities were far too sparse for the insects to have maintained the alignments by visual reference to one another [5], and so the orientation patterns must have been due to individual responses to some environmental cue or cues. The obvious means for a flying insect to determine the wind direction is by the visual perception of the apparent movement of the ground, in a similar way to the optomotor response which occurs in flight near the ground [6]–[7]. However, evidence has steadily accumulated that suggests that, in some cases at least, a visually-mediated optomotor-type mechanism is not the predominant cue [8], and that high-altitude orientation may be maintained by some intrinsic feature of the wind itself [8], [9]. Reynolds et al. [8] hypothesised that shear-associated turbulence directionality coupled with inertial lag of the insect mass can provide cues for common orientation and there was some evidence for this in the offset of heading directions from the downwind direction in ‘medium-sized’ (10–70 mg) insects. Larger moths (>100 mg) adopt a different strategy; they migrate only when the mean wind direction is in, or near, a seasonally advantageous direction – in other words they orientate with respect to the wind *and* have a compass sense [10]–[12]. Here we examine a previously overlooked but plausible alternative hypothesis − that high-flying migrant insects use ‘temperature ramps’ as orientation cues, before going on to reconsider ways in which turbulent fluctuations in wind velocity or acceleration might indicate the mean wind direction.

**Figure 1:**
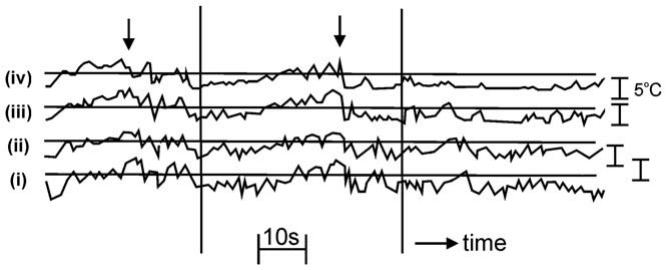
Some typical time traces of the temperature fluctuations in the atmosphere, showing characteristic ramp-like features. The recordings were made by Phong-Anant *et al.* [14] by the simultaneous use of four temperature probes at heights of (i) 1 m, (ii) 2 m, (iii) 4 m and (iv) 8 m above the ground. The probe at 4 m was 1 m upwind of the others. Using Taylor's hypothesis (i.e. assuming that turbulence is frozen in time and advected downwind by the mean flow), the time traces are equivalent to taking spatial cuts by a fast-moving probe. Arrows indicate two examples of sharp structures.

Records of temperature within the atmospheric boundary-layer show characteristic ‘ramp-cliff’ structures [13]–[17]. In the first 8 m of a stable atmospheric surface layer, for example, temperature ramps last about 10 to 20 s during which temperature can increase by several degrees [14] (Figure 1). Ramp-cliff structures have also been observed in turbulent jets [18], [19] which are of interest because common orientation is often seen in insect layers that form in low-level nocturnal jets.

Ramp-cliff structures are evident in the downwind direction but not the crosswind direction, and therefore airborne insects could use these ramps to orientate downwind. This could be achieved by their detecting near-periodic fluctuations in temperature when flying downwind, and more random fluctuations in temperature when flying in other directions.

## Methods

The degree of common orientation can be expected to increase with the strength of the orientation cue. The size of the temperature ramps is expected to increase as the gradients in mean wind speed and mean temperature increase, and it follows that if insects do use temperature ramps as orientation cues then the mean gradient in these two variables should be accompanied by an increase in the ‘tightness’ of orientation (i.e. a decrease in angular dispersion around the mean value). To look for evidence of this in nocturnal insect migrants, we used data from two specially-developed vertical-looking entomological radars (VLRs) located in the southern UK that provide information on the flight characteristics of individual, high-altitude, insect migrants [20]–[22]. Overflying insects can be simultaneously detected within 15 different height bands (‘range gates’ of 45 m depth), between ∼150 m and 1200 m above the VLRs, and the displacement direction and body alignment of each individual is routinely recorded. This enabled us to examine the orientation behaviour of medium-sized (<80 mg) and large-sized (>80 mg) nocturnal insects migrating high above the ground, in relation to the wind currents in which they were flying. Nocturnal migrants in the medium size class are likely to be predominantly composed of larger ‘micro’-Lepidoptera (e.g. including migrant pyralids such as *Nomophila noctuella* and *Udea ferrugalis*); some small noctuid moths; green lacewings (Neuroptera); and certain families of Diptera and Coleoptera [22]–[28]. The large size class will predominantly be composed of migrant noctuid moths such as *Autographa gamma* and *Noctua pronuba* [10]–[12].

We used linear regression to test whether there was any evidence that either gradients in mean temperature or mean wind-speed had an effect on the ‘tightness’ of nocturnal insect common orientation, as measured by the vector length “R”, of the flight headings of a group of insects that showed significant levels of common orientation in the first place. To do this we made use of data from vertical-looking radars for the flight patterns of nocturnal migrating insects, and meteorological data from the UK Meteorological Office's “Unified Model” (UM). Initially, this was done for 53 nocturnal migration ‘events’ involving large insects (>80 mg) flying during a 1-hour period and across a number of consecutive radar gates (typically about 5 or 6, spanning ∼400 m vertical range). UM data from the midpoint of the time-span, and from UM heights near to the top and bottom of the radar height-range were selected for each event. The analysis was then repeated for ‘medium-sized’ nocturnal insects (<80 mg) flying in 1-hour periods at a particular height interval during 50 migration ‘events’, which corresponded almost exactly to the 53 events for ‘large’ insects. Linear regression analyses were carried out using Genstat® [29] statistical analysis package.

## Results

### Temperature ramps as putative indicators of the mean wind direction

**Figure 2:**
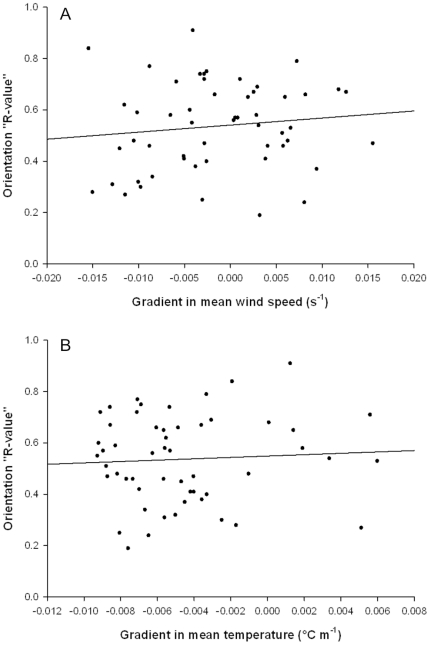
The ‘tightness’ of orientation direction in large insects and gradients in wind speed and temperature. Linear regression analyses for N = 53 nocturnal ‘events’ (see text) composed of large insects (>80 mg) (**A**) Orientation ‘R-value’ and gradient in mean wind speed shear: df  = 1, 51, R^2^<0.001, F = 0.75, P = 0.389. (**B**) Orientation ‘R-value’ and gradient in the mean temperature: df  = 1, 51, R^2^<0.001, F = 0.20, P = 0.660.

**Figure 3:**
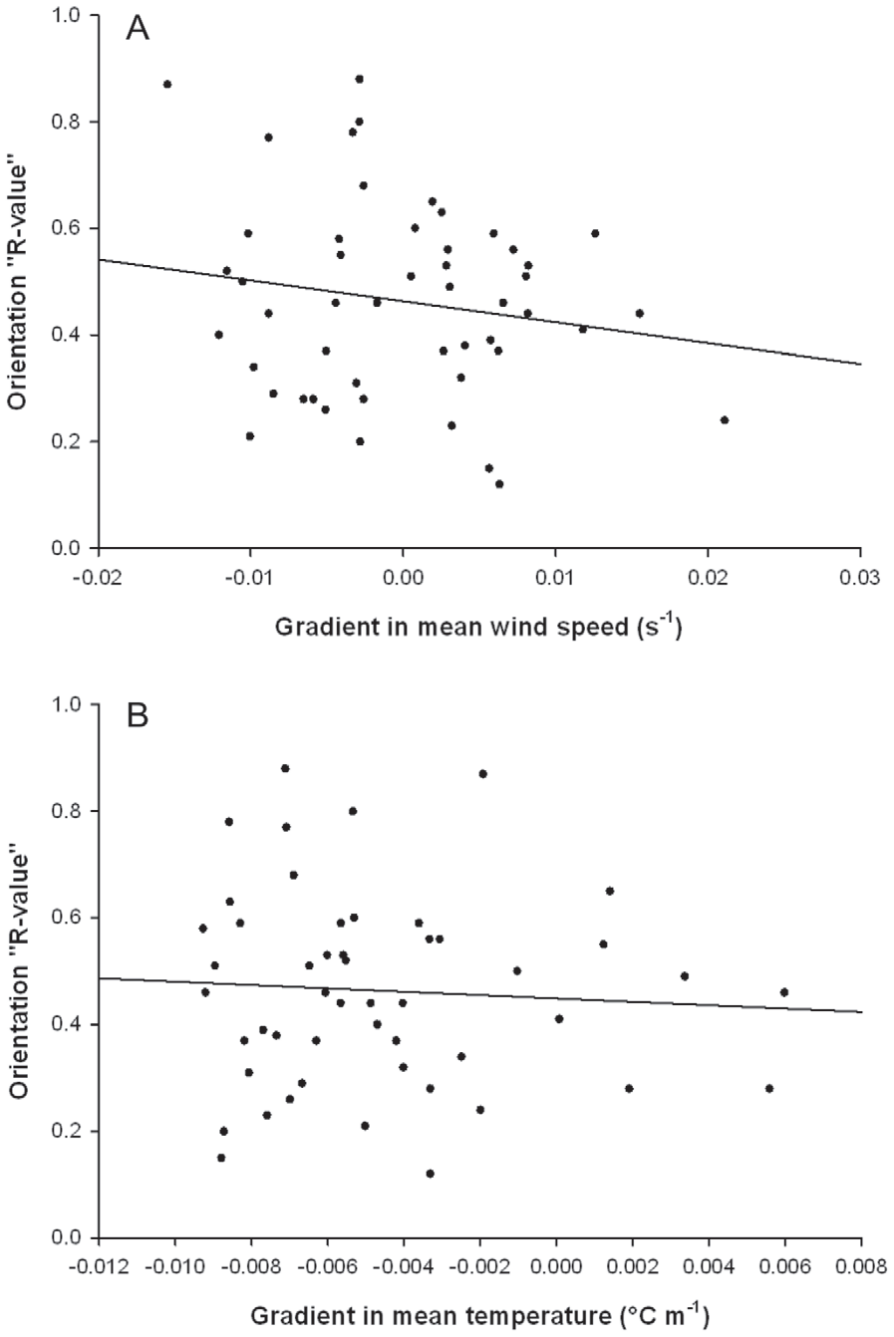
The ‘tightness’ of orientation direction in small insects and gradients in wind speed and temperature. Linear regression analyses for N = 50 nocturnal ‘events’ of medium-sized insects (<80 mg). (**A**) Orientation ‘R-value’ and gradient in mean wind speed shear: df  = 1, 48, R^2^<0.001, F = 1.38, P = 0.246. (**B**) Orientation ‘R-value’ and gradient in the mean temperature: df  = 1, 48, R^2^<0.001, F = 0.21, P = 0.652.

We found no evidence that gradients in either mean temperature or mean wind-speed shear showed any relationship with the degree of common orientation (Figures 2 and 3). Similarly, we found no evidence that absolute gradients (where we disregarded the signs) were related to the degree of common orientation. We also carried out multiple regressions of gradient in mean temperature and mean wind-speed on the tightness of common orientation, and found that all possible combinations were non-significant.

Our analysis suggests, incidentally, that gradients in chemical concentration (odour cues), which might conceivably be thought to influence the degree of common orientation, can also be excluded as a mechanism. This is because gradients in chemical concentration and temperature are aligned, as the Schmidt number (the ratio of the dynamic viscosity and mass diffusivity) of most chemicals in air is close to unity.

### Turbulent fluctuations in velocity and acceleration as putative indicators of the mean wind direction

Here we elaborate on our previous theoretical analysis of Reynolds et al. [8] to uncover further ways in which turbulent fluctuations in velocity and/or accelerations can provide indications of the mean wind direction. Our previous analysis accounted explicitly for spatial gradients in turbulent velocity statistics but was restricted in applicability to rather small insects (with aerodynamic response times much less than the timescale on which turbulent fluctuations in wind speed are correlated). The new analysis is applicable in the absence of spatial gradients in turbulent velocities and applies to both small and large insects. The theoretical predictions are in qualitative agreement with the results (not shown) of numerical studies using ‘kinematic simulations’ [30] of low Reynolds number 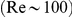 homogeneous turbulence by unsteady random Fourier modes. In contrast with the theory, these simulations take explicit account of turbulent-flow structures, incompressibility of the air flow, preferential concentration into regions of the flow with high strain [31] and the peculiar statistical properties of air-flows along particle trajectories which are neither purely Lagrangian (fluid-particle like) nor purely Eulerian-like [32]. Suppose for simplicity that the turbulent velocity fluctuations experienced by an insect average to zero, are Gaussian, homogeneous and at the smallest scales isotropic. The simplest model encapsulating this scenario is the classic Langevin equation,
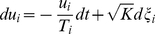
(1)where: 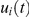 is the velocity of the air at the current location of the insect; the indices denote Cartesian coordinates; 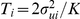
are the autocorrelation time scales; 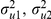 and 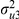 are the velocity variances for fluctuations in streamwise, crosswind and vertical directions; 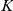 is a constant and; 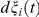 are an incremental Wiener process with 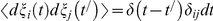. In the simplest case, insects could behave like passively advected particles subject to Stokes' drag law,
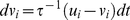
(2)where 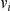 is the velocity of the insect and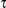 is the Stokes aerodynamic response time. This is the so-called ‘oscillating Stokes problem’. A straightforward calculation using Eqns. (1) and (2) reveals that the mean air velocity (relative to the ground) experienced by particles moving with instantaneous velocity 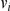, 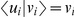 where the angular brackets denote an average over all possible air velocities. The conditional mean air velocity (i.e. the mean air velocity for a given particle velocity) is just the instantaneous particle velocity, and it follows that the unconditional mean air velocity 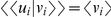 where the outer angular brackets denote an average over all possible particle velocities. As expected the mean particle velocity is just the mean air velocity. There are, however, significant differences between the instantaneous air- and particle-velocities. Mean-square differences between the air and particle velocities, 
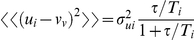
. These differences vanish for tracer-particles 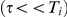. Particles with very high inertia 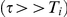 barely respond to turbulent gusts in air velocity so that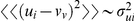. Thus turbulent air gusts will typically be moving past larger (∼50 mg) insects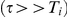 and these may be discernible. The gusts provide an indication of the mean wind direction as 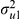 is typically larger than 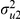 and 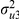. Larger insects that aligned themselves in the direction of the strongest gusts would be orientated in either an upwind or downwind direction. The 180° ambiguity in the determination of the mean winddirection is resolved when there is a turbulent shear-stress, i.e. when 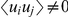 for 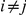, because there is then a prevalence for gusts with a positive streamwise component to have either an upward or downward component and there is a prevalence for gusts from the left of the mean wind line when the Ekman spiral is in full effect. Insects which orientate themselves to minimise this sidewise buffeting will when flying in the northern hemisphere be offset to the right of the mean wind line. Such offsets have recently been observed [8]. It can also be shown that particle (insect) accelerations have a mean of zero and variance 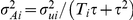. For small insects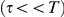, the acceleration variances reduce to 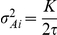, i.e. smaller insects experience isotropic accelerations that provide no indication of the mean wind direction. Larger insects 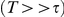, on the other hand, experience anisotropic accelerations, 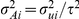 and these may be discernible and used as orientation cues if 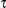 is not too large.

## Discussion

The environmental cues used by nocturnal insect migrants to select and maintain common headings, while flying at altitudes of several hundreds of metres above the ground in low illumination levels, and the adaptive benefits of this behaviour, have long remained a mystery. There is now mounting evidence that high-altitude nocturnal insect migrants are using turbulent features of the wind flow rather than visual cues to orientate to the mean wind direction [8]. Here we examined and discounted the possibility that airborne insects are using turbulent fluctuations in air temperature as a cue.

If insects are ignoring this apparently evident cue, this bolsters the case for velocity fluctuations due to atmospheric turbulence being the predominant orientation cue. Previously we showed how such turbulent cues can explain both high-altitude orientation to the mean wind direction and layering of rather small nocturnally-migrating insects [8]. Here we extend the theory to encompass both small and large insects and show that the turbulent velocity fluctuations and accelerations felt by the large insects are predicted to be anisotropic even when the small-scale turbulence (measured at a fixed point, or along the trajectory of a fluid-particle) is isotropic. The mechanism(s) by which airborne insects actually sense these small air flows remain to be elucidated. Antennae could be the mechano-receptors responsible for detecting the weak air-flows through their action on Johnston's organ [33]–[35]. The mechanism could also involve differential measurements of air pressure on either side of the body, linear or angular acceleration, or proprioceptors in the wings. But antennae are more likely because they are held out in front of the insect, and so may be better at compensating turbulent cues against the ‘noise’ due to wing beats. Turbulent flow past the antennae could, for example, cause vortex shedding from alternate sides that create periodic lateral forces, causing the antennae to vibrate [36], forming the basis for a velocity sensor. Vortex shedding velocity sensors have, in fact, found widespread application in commercial flow meters. We anticipate that a better understanding and quantification of the turbulent fluctuations in velocity and acceleration experienced by insects will come from experimental studies that track particles in laboratory flows with high Reynolds number and report on measurements of particle accelerations and velocities [37]–[40].
